# Reliability of a script agreement test for undergraduate speech-language therapy students

**DOI:** 10.1590/2317-1782/20232022098en

**Published:** 2023-11-13

**Authors:** Angélica Pilar Silva Ríos, Manuel Nibaldo del Campo Rivas, Patricia Katherine Kuncar Uarac, Víctor Antonio Calvo Sprovera

**Affiliations:** 1 Escuela de Fonoaudiología, Facultad de Salud, Universidad Santo Tomás - Chile.; 2 Centro Interdisciplinario de Innovación Educativa, Universidad Santo Tomás - Chile.; 3 Escuela de Fonoaudiología, Facultad de Ciencias de la Salud, Universidad Católica Silva Henríquez - Santiago, Región Metropolitana, Chile.

**Keywords:** Reliability, Reproducibility of Results, Higher Education, Speech-Language Pathology, Professional Competence

## Abstract

**Purpose:**

To estimate the reliability of scripts designed for undergraduate Speech-Language Therapy students.

**Methods:**

A descriptive cross-sectional study was carried out. Qualitative variables were summarized by frequency or proportion and quantitative through means (CI 95%). Reliability was estimated through Cronbach's α coefficient, and inter-rater agreement was determined using Fleiss’s Kappa index. The analytical tests considered a significance level of p<0.05.

**Results:**

80 scripts organized in four areas of speech-language therapy were validated by 41 speech-language pathologists. The average experience of the professionals was 17.1 years. The reliability of the corpus was α: 0.67 (min= 0.34; max: 0.84), and the inter-rater agreement κ: 0.29 (min: 0.07; max: 0.45).

**Conclusion:**

The corpus's reliability scores were similar to those reported by previous studies in different health professions. Having validated strategies aimed at developing proficiency and supporting classic training actions in undergraduate courses will contribute to increasing the quality of future health professionals.

## INTRODUCTION

The training of health professionals has become more complex to the extent that disciplinary and teaching knowledge has expanded, involving teaching-learning processes supported by evidence, centered on the student, and accompanied by the use of technologies, innovation, and high scientific standards^(1)^. Students must develop proficiency involving knowledge, skills, behaviors, and values that allow them to approach users in diverse and uncertain contexts^(2)^.

Among the skills that students must develop in their academic training, there is clinical reasoning (CR), a relevant component for developing competencies that allow inferring a diagnosis and choosing the most appropriate routes of action for each user^(3)^. In the case of Speech-Language Therapy, a discipline in charge of addressing and preventing communication and swallowing disorders^(4)^, undergraduate training requires the student to develop complex skills^(5)^, among which is the CR, a challenging competence in terms of professional training^(6)^. Despite the existence of heterogeneous definitions, it is accepted that CR implies using skills such as: analysis, problem-solving, contextual reasoning, and pattern recognition^(7)^. Such tasks seem to happen automatically in the case of experts. However, novice students require training to achieve them^(8)^.

This way, CR is considered a fundamental skill in the approach to users, which must be incorporated into the study plans of health professionals. Within the strategies described in the literature, using scripts (clinical scripts) has offered a framework to understand how thinking is organized when training CR^(9)^.

The scripts respond to schemes associated with sequences of events that occur in a particular order^(10)^, organizing themselves as a set of attributes that are more or less likely to happen and that contain symptoms and signs that are linked, forming generic structures that can represent any disease^(9)^. This is how the scripts represent a decision-making model based on hypothesis generation. These are raised based on user signs and data that allow confirming or rejecting these assumptions through psychological or mental processes in which theoretical knowledge and accumulated practical experience participate^(9)^.

In this line, different studies have shown the effectiveness of scripts as a tool for teaching CR skills^(11)^ and as a valid and reliable evaluation tool^(12)^.

It has been observed that the evaluation methods traditionally used to measure competencies are limited in assessing CR^(13)^. This is how the script agreement test has been described as an appropriate tool for training the CR, since it allows exercising decision-making and data interpretation in situations of uncertainty^(14)^. The scripts enable students to assess the change in the probability of a hypothesis, contrasted with new information, in scenarios focused on making diagnostic, study, or intervention decisions. These hypotheses are evaluated on a 5-point Likert scale, with positive, neutral, or negative response options^(15)^. A methodology challenge points out that there is still little information on cut scores or their correlation with knowledge scores at the expert level. On the other hand, the literature highlights that experienced professionals tend to avoid extreme responses offered by the script, increasing test scores^(16)^, with the consequent probability of generating biases in its construct^(17)^. The script agreement test has been used in different health professions as a strategy for CR training; however, no studies validate its use in Speech-Language Therapy, which requires the development of scripts relevant to the profession and the need to determine specific psychometric properties. This research aimed to estimate the reliability of a corpus of clinical scripts designed for undergraduate Speech-Language Therapy students.

## METHODS

The Scientific Ethics Committee of Santo Tomás University approved the research with code 153.20. Each participant signed an informed consent to enter the project.

A descriptive study of transverse cut. The universe was made up of professionals who worked in various clinical fields of speech-language therapy and had a degree in Speech-Language Therapy granted by a Chilean or foreign university. Sampling was done for convenience following the snowball strategy. The inclusion criteria were: having a minimum of 3 years of professional experience in the disciplinary area, having a Master's or Doctor's degree, and being authorized as an individual provider in the National Registry of the Health Superintendence (HS). The exclusion criteria were: working preferably in management or teaching tasks (without clinical activity) and being currently inactive in labor terms due to retirement. The necessary number of experts was selected according to the recommendation of the literature for the validation of clinical scripts, estimating the incorporation of at least ten professionals per area^(18)^. The intervened career contemplates four core areas, namely: Child-Youth Communication and Language; Voice and Orofacial Motor Skills; Cognition and Communication in Adults and the Elderly; Audiology and Vestibular, for which the minimum n was projected in 40 speech-language therapists who were contacted at the national level through the sampling technique mentioned earlier, corroborating an intermediate professional experience, and with a gender distribution consistent with the professional profile of the practitioners in the country.

In procedural terms and for creating the corpus, eight professionals (two per area) were invited to write the scripts to train and evaluate CR in diagnostic, study, and intervention tasks. These experts received synchronous training of three chronological hours, made online, through the Microsoft Teams platform.

The training was facilitated by an academic with a doctorate in education and applied experience in the methodology, specifically for undergraduate and postgraduate medical training. Two speech-language therapists with clinical and educational experience advised the instance, belonging to the original undergraduate program. A theoretical presentation was made, followed by a workshop for creating scripts in virtual rooms according to area or field of training. Subsequently, the products were socialized, and feedback was provided by the facilitator to solve any procedural or theoretical doubts concerning the preparation of scripts. Each professional participating in the training was responsible for creating ten scripts.

Following the methodology reported in the literature, the population that would use the clinical scripts was determined^(19)^; in this case: students who completed the seventh and eighth semesters of the Speech-Language Therapy course at Santo Tomás University, at the Viña del Mar, Santiago, and Puerto Montt campuses, during the year 2021. For this research, the students did not use the scripts; however, their characterization is important, given their relevance as recipients of the final product. The identification of key contents for the corpus was made by analyzing learning results (LR) of subject programs^(20)^ that contributed to the four areas previously declared.

A corpus of 80 scripts was created, organized into four training areas, meaning 20 per area. The scripts for each area were classified as follows: eight diagnostic scripts, two study scripts, and ten intervention scripts.

Once the scripts were generated in the established format, they were submitted to be reviewed by the research team made up of two speech-language therapists with master's degrees and experience in teaching and educational innovation, together with the expert with a doctorate degree from the medical training area, to standardize the texts and adapt them to the purposes of the study.

For the response and validation of the clinical scripts (80 in total), each Speech-Language therapist received a booklet with instructions and had to respond within a maximum of 2 weeks, depending on their area of expertise.

The responses of the speech pathologists were tabulated in Microsoft Excel. The response frequency was determined for descriptive purposes by the script to calculate the score later, transformed score, and final score according to the previously reported methodology. Quantitative variables were summarized by measures of central tendency and dispersion, considering a CI of 95%. According to the recommendation of the literature^(21)^, the internal consistency was estimated from Cronbach's α coefficient, and inter-observer reliability was calculated with Fleiss's Kappa coefficient, considering a significance level of p<0.05^(22)^. Responses were stratified by area, type of script, and type of response to control confounding variables. The incomplete scripts that presented very low response frequencies according to the outliers method were eliminated from the corpus^(23)^.

The statistical analysis was performed in Stata Statistical Software: Release 15. College Station, TX: StataCorp LLC.software.

## RESULTS

Forty-one speech pathologists answered the booklets delivered by area, with a minimum age of 28 years and a maximum of 61. Regarding the distribution by gender, 78% of the women were observed in the field of Child-Youth Communication and Language, and 60% of the males in Voice and Orofacial Motor Skills. 98% of the informants had a master's degree and 2% a doctorate. The average professional experience was recorded in a range of 10.6 to 17.2 years (Table 1).

**Table 1:** Sociodemographic characteristics of the experts by area

**Area**	**Experts**	**Age**			**Experience**			**Gender**	
	n	Average	Standard Error	Reliability Interval 95%	Average	Standard Error	Reliability Interval 95%	Male	Female
**Child-Youth Communication and Language**	11	40.7	2.56	35 - 46.45	17.18	2.45	11.7 - 22.66	22%	78%
**Voice and Orofacial Motor Skills**	10	35.4	2.16	30.51- 40.29	11.2	2.22	6.17 - 16.22	60%	40%
**Cognition and Communication in Adults and the Elderly**	10	34	0.82	32.15 - 35.8	10.6	0.76	8.87 - 12.32	40%	60%
**Audiology and Vestibular**	10	34.8	1.38	31.66 - 37.94	10.6	1.31	7.62 - 13.58	40%	60%

Caption: n = number of participants

**Figure 1:**
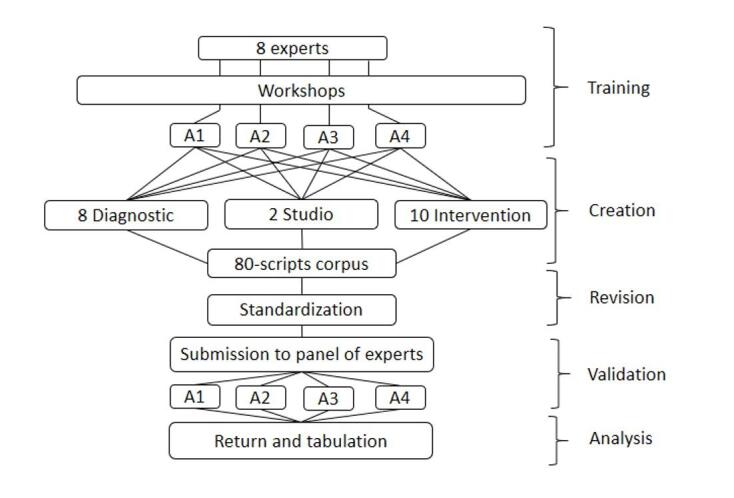
Procedure for the validation of the Scripts corpus

**Figure 2:**
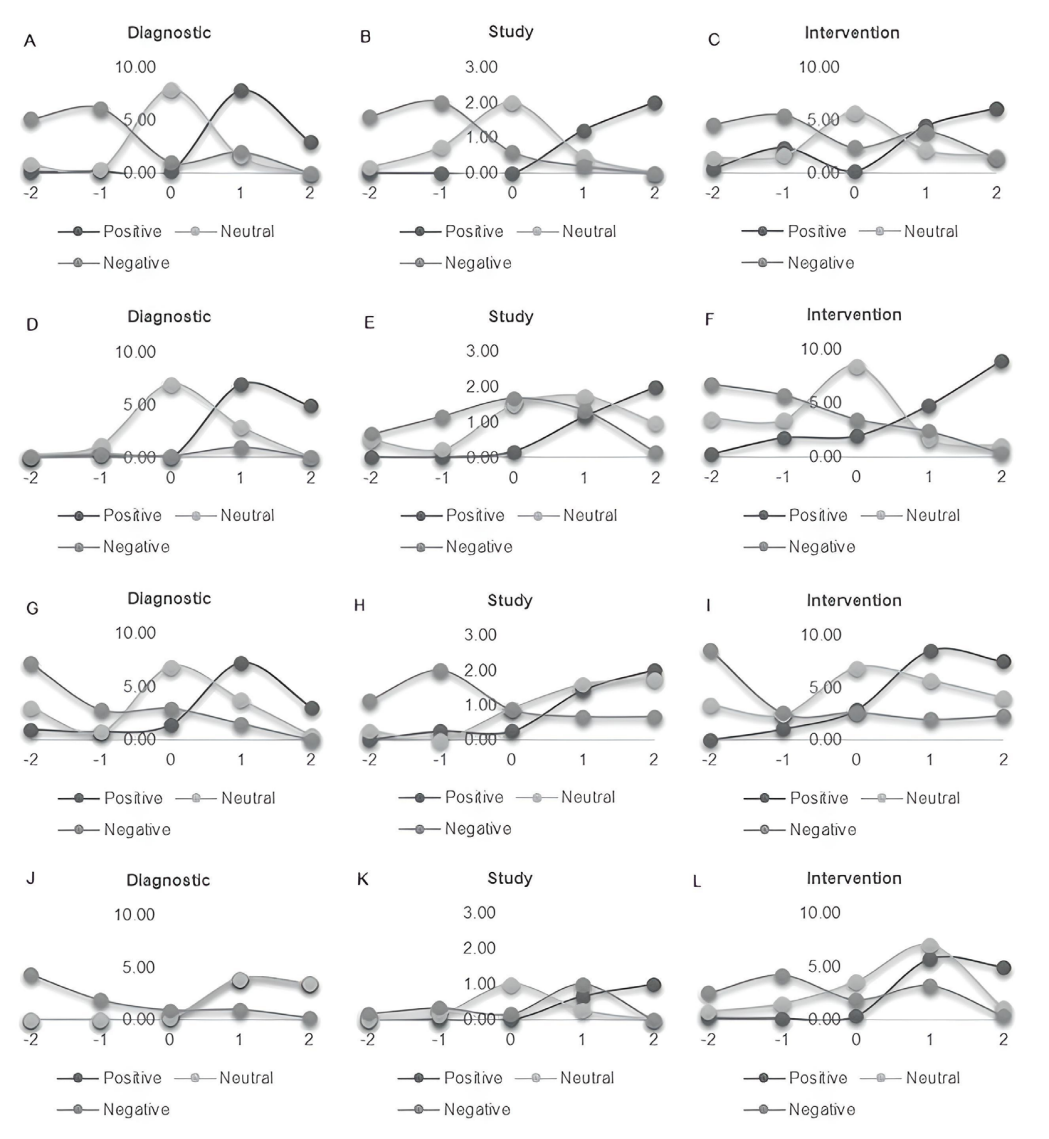
Final scores by area and type of script

The final scores added and stratified by area and type of script reveal consistent response trends among practitioners (Figure 1). It is evident that the frequency of the added scores is usually adjusted to the type of response expected by the script: positive, neutral, or negative. In the case of the areas of Voice and Orofacial Motor Skills, together with Cognition and Communication in Adults and the Elderly, a shift towards positive values of neutral responses was noted (Figure 2E, H) for study-type scripts, and in the case of diagnosis (Figure 2J), and intervention and Audiology and Vestibular scripts (Figure 2L). In the case of the Voice and Orofacial Motor Skills area, in the same way, there is a movement of the negative responses to the right for the diagnostic case (Figure 2D) and study (Figure 2E) and the same situation was observed in the study scripts in the Audiology and Vestibular area (Figure 2K).

Caption: A1 = Child-Youth Communication and Language; A2 = Voice and Orofacial Motor Skills; A3 = Cognition and Communication in Adults and the Elderly; A4 = Audiology and Vestibular

Caption: A-C = Child-Youth Communication and Language; D-F = Voice and Orofacial Motor Skills; G-I = Cognition and Communication in Adults and the Elderly; J-L = Audiology and Vestibular

The internal consistency of the corpus was α= 0.67. When analyzing by area and type of Script, low reliability was observed (α=0.60-0.70) for the diagnostic items in the four areas and the intervention scripts in the case of Cognition and Communication in Adults and the Elderly. It should be noted that insufficient reliability was recorded in the Audiology and Vestibular area case for the study scripts (α=0.34). On the other hand, the scripts of the same type in the remaining areas showed acceptable reliability (α=0.80-0.90) (Table 2).

**Table 2:** Corpus Reliability by area and type of scripts

**Area**	**Type of Script**	**α Cronbach**
**Child-Youth Communication and Language**	Diagnostic	0.66
	Study	0.84
	Intervention	0.59
**Voice and Orofacial Motor Skills**	Diagnostic	0.68
	Study	0.82
	Intervention	0.72
**Cognition and Communication in Adults and the Elderly**	Diagnostic	0.69
	Study	0.82
	Intervention	0.65
**Audiology and Vestibular**	Diagnostic	0.69
	Study	0.34
	Intervention	0.64

The agreement between evaluators was estimated at κ= 0.29 (acceptable) for the corpus. When stratifying according to the variables of area and type of script, a slight concordance was observed in the areas of Voice and Orofacial Motor Skills (κ= 0.07), Cognition and Communication in Adults and the Elderly (κ= 0.08). The rest of the items presented an acceptable agreement (κ>0.20 and <0.41) and moderate agreement for the diagnostic scripts in Audiology and Vestibular (κ= 0.45) (Table 3).

**Table 3:** Fleiss’s Kappa Index by area and type of Script

**Area**	**Type**	**Combined Kappa**	**Z**	**Prob > Z**
**Child-Youth Communication and Language**	Diagnostic	0.38	25.43	0.0001
	Study	0.25	8.99	0.0001
	Intervention	0.21	13.85	0.0001
**Voice and Orofacial Motor Skills**	Diagnostic	0.39	24.42	0.0001
	Study	0.07	2.07	0.0193
	Intervention	0.27	19.64	0.0001
**Cognition and Communication in Adults and the Elderly**	Diagnostic	0.26	15.51	0.0001
	Study	0.08	2.41	0.008
	Intervention	0.17	12.2	0.0001
**Audiology and Vestibular**	Diagnostic	0.45	22.76	0.0001
	Study	0.22	4.27	0.0001
	Intervention	0.22	12.47	0.0001

## DISCUSSION

The diversification of teaching strategies oriented to the development of complex competencies such as CR, is key in training undergraduate health students^(24)^. To date, experiences implementing the script agreement test have been reported in careers such as medicine and nursing^(25)^, among others. The results of this study show variability in the judgment of the experts by area (Figure 1), which is aligned with the description of the decision-making process in uncertainty scenarios^(26)^. It is consistent with previous research experiences where divergent responses have been described as a recurring finding in expert panels' resolution of clinical scripts^(23)^.

The evaluation of the CR is a complex process in pedagogical terms^(27)^, and the scripts have proven to be a reliable and valid teaching tool when there is a rigorous design and build process^(28)^. In this sense, it is essential to highlight that the scripts have made it possible to discriminate differential degrees of performance according to the educational level and the time of professional experience in medicine^(28)^; hence its application for the postgraduate course in speech-language therapy represents an interesting projection that can be explored in future research.

The use of scripts implies certain challenges when thinking about their design, construction, and implementation, specifically related to the validity of the corpora, the handling of low scores, and the association of these results with other evaluation techniques. In this sense, and for practical purposes, as reported in the methodology, all the scripts that presented incomplete answers and those that did not have dominant agreements were eliminated from the corpus, that is, a considerably low frequency considered as such by the experts^(23)^. Thus, a global reliability of α= 0.67 was obtained, which, although considered low, is similar to what was reported in a previous study in postgraduate medicine (α>0.65 in all items)^(29)^.

The consistency in the experts’ answers to the scripts, in general, is acceptable beyond chance (Table 3), except in study scripts in the areas of Voice and Orofacial Motor Skills, Cognition and Communication in Adults and the Elderly. This finding is consistent in relation to the studies in other disciplines where there is a coincidence in the agreement between the validators with scores that are usually higher than those observed in novices without experience, and allow discriminating the educational level and proficiency^(30)^. In this way, it is verified that the dossier created has properties which make it functional for evaluating CR skills in Speech Pathology students, as the evidence highlights it in other areas of health^(29)^.

A limitation of this study is the variability in what is done and the professional judgment, which is expressed as the dispersion in response trends by experts in certain areas (Figure 2D, E, H, J) and has an impact on the reliability scores (Table 2). However, the literature offers some explanations for this phenomenon, such as the tendency of validation professionals to avoid extreme responses, that is, -2 or +2 in scripts^(16)^. In this line, although the overall reliability score was low, it is important to highlight that the poor reliability result (α= 0.34) was maintained in the analysis in the case of the study scripts in the Audiology and Vestibular area, which affected the final calculation on the dossier on the one hand but avoided the confirmatory bias. Yet, the discrepancy between the experts is acceptable, considering that the validated instrument does not correspond to a diagnostic instrument.

Concerning the sample size, it is important to note that the literature recommendation was considered, so although it is possible to expand the number of experts per field, this research adhered to what was suggested by previous research^(18)^.

As a projection, it is interesting to incorporate validation of the content of the dossier beyond the ascription to the learning results of the subject programs, as it is to add a second evaluative instance to determine the consistency of the intra-evaluator responses, increase the number of scripts per type, and increase the number of validators. Likewise, it will be essential to determine the impact and association of incorporating this strategy with respect to students' progress beyond clinical decision-making in scenarios with high uncertainty^(31)^.

## CONCLUSION

The corpus created presented a low global reliability. Still, the stratification by area and type of script offers a heterogeneous panorama, where it is possible to distinguish those items where a greater or lesser correlation was obtained, depending on the clinical reasoning task presented. Additionally, the inter-evaluator agreement was acceptable for the entire dossier. Determining the psychometric properties of scripts in the context of the script agreement test is the first formal approach to generating an innovative pedagogical tool for the training and authentic evaluation of CR in undergraduate students of the Speech-Language Therapy career. It is necessary to increase research and educational innovation in this field to offer relevant training instruments which are based on available scientific evidence and have validation to improve the development, monitoring and evaluation of professional proficiency in students of health careers, in preclinical instances and/or in early formative stages, complementing the strategies, instruments and tools already established in the curricula. The strategy developed is transferable, but it must be adjusted to the curricular frameworks of each house of study in terms of content and learning outcomes.
