# Supplementary material for: Reliability of a script agreement test for undergraduate speech-language therapy students
Source: Codas. 2023 Nov 13;35(5):e20220098. doi: 10.1590/2317-1782/20232022098en (PMC10688298; doi:10.1590/2317-1782/20232022098en)
Supplement: Translation

# Translation: Validación de constructo y reproducibilidad de una prueba de concordancia de guiones para estudiantes de pregrado en fonoaudiología

**DOI:** 10.1590/2317-1782/20232022098es

**Authors:** Angélica Pilar Silva Ríos, Manuel Nibaldo del Campo Rivas, Patricia Katherine Kuncar Uarac, Víctor Antonio Calvo Sprovera

**Affiliations:** 1 Escuela de Fonoaudiología, Facultad de Salud, Universidad Santo Tomás - Chile.; 2 Centro Interdisciplinario de Innovación Educativa, Universidad Santo Tomás - Chile.; 3 Escuela de Fonoaudiología, Facultad de Ciencias de la Salud, Universidad Católica Silva Henríquez - Santiago, Región Metropolitana, Chile.

**Keywords:** es: Confiabilidad, Reproducibilidad de los Resultados, Educación Superior, Fonoaudiología, Competencia Profesional

## Abstract

**Objetivo:**

Estimar la confiabilidad y reproducibilidad de un corpus de scripts diseñado para el pregrado de fonoaudiología.

**Método:**

Estudio observacional de tipo descriptivo y temporalidad transversal. Se estimó la validez de constructo a partir del coeficiente α de Cronbach y la reproducibilidad con el índice Kappa de Fleiss. Las pruebas analíticas consideraron un nivel de significancia p<0.05.

**Resultados:**

Se creó un corpus de 80 scripts organizados en 4 áreas de la fonoaudiología el que fue validado por 41 fonoaudiólogos. La experiencia promedio de los profesionales fue de 17.1 años (Std. Err: 2.4; IC 95%: 11.7-22.6). La confiabilidad del corpus fue α: 0.67 y el acuerdo interevaluador, κ: 0.29.

**Conclusión:**

Los puntajes de confiabilidad y reproducibilidad del corpus creado fueron similares a los reportados por estudios previos en otras profesiones de la salud. Contar con estrategias validadas que se orienten al desarrollo de competencias y complementen las acciones formativas, contribuirá a incrementar la calidad en la formación de futuros profesionales de la salud.

## INTRODUCCIÓN

La formación de profesionales de la salud se ha complejizado en la medida en que se amplía el conocimiento disciplinar y docente, involucrando procesos de enseñanza - aprendizaje sustentados por la evidencia, centrados en el estudiante y acompañados del uso de tecnologías, innovación y elevados estándares científicos^(1)^. En este contexto, los estudiantes deben desarrollar competencias que implican conocimientos, habilidades, comportamientos y valores que les permitan abordar usuarios en diversos e inciertos escenarios^(2)^.

Entre las destrezas que el estudiantado debe desarrollar en su formación académica, se encuentra el razonamiento clínico (RC), componente relevante para el desarrollo de competencias que permiten inferir un diagnóstico y elegir las rutas de acción más adecuadas a cada usuario^(3)^. En el caso de la fonoaudiología, disciplina encargada del abordaje y prevención de trastornos de la comunicación y deglución^(4)^, la formación de pregrado requiere que el estudiante desarrolle destrezas complejas^(5)^ entre ellas, el RC, competencia retadora en lo que a la formación profesional respecta^(6)^. A pesar de existir definiciones heterogéneas, se acepta que el RC implica el uso de habilidades cognitivas como: análisis, resolución de problemas, razonamiento contextual y reconocimiento de patrones^(7)^. Dichas tareas parecen ocurrir de manera automática en el caso de los expertos, sin embargo, los estudiantes novatos requieren de entrenamiento para su alcance^(8)^.

De esta forma, se considera al RC como una habilidad fundamental en el abordaje de usuarios, que debe ser incorporada en los planes de estudio de profesionales de la salud. Dentro de las estrategias descritas en la literatura, el uso de scripts (guiones clínicos), ha ofrecido un marco para comprender cómo se organiza el pensamiento a la hora de entrenar el RC^(9)^.

Los scripts responden a esquemas asociados con secuencias de eventos que ocurren en un orden determinado^(10)^, organizándose como un conjunto de atributos que tienen más o menos probabilidad de ocurrir y que contienen síntomas y signos que se vinculan, conformando estructuras genéricas que pueden representar cualquier enfermedad^(9)^. Es así como los guiones estructuran un modelo de toma de decisiones basado en la generación de hipótesis. El guión es planteado a partir de señales y síntomas de los usuarios que estructuran una viñeta de alta incertidumbre, tres escenarios posibles y datos adicionales que permiten confirmar o desechar dichas suposiciones a través de procesos psicológicos o mentales, en los que participa el conocimiento teórico y la experiencia práctica acumulada^(9)^.

En esta línea, diversos estudios han demostrado la eficacia de los scripts como herramienta para enseñar habilidades de RC^(11)^ y a su vez como herramienta de evaluación válida y fiable^(12)^.

Se ha observado que los métodos de evaluación que tradicionalmente se utilizan para la medición de competencias son limitados en lo concerniente a la valoración del RC^(13)^, es así como la prueba de concordancia de guiones (scripts) ha sido descrita como una herramienta apropiada para entrenar el RC, ya que permite ejercitar la toma de decisiones y la interpretación de datos en situaciones de incertidumbre^(14)^. Los scripts permiten que los estudiantes valoren el cambio en la probabilidad de una hipótesis, contrastada con nueva información, en escenarios enfocados en la toma de decisiones diagnósticas, de estudio o intervención. Dichas hipótesis se evalúan en una escala Likert de 5 puntos con opciones de respuesta positiva, neutra o negativa^(15)^. Un desafío de la metodología señala que aún existe poca información sobre los puntajes de corte o su correlación con los puntajes de conocimiento a nivel de expertos. Por otra parte, la literatura destaca que los profesionales con experiencia tienden a evitar las respuestas extremas ofrecidas por el guión, aumentando los puntajes de las pruebas^(16)^, con la consecuente probabilidad de generar sesgos en su constructo^(17)^. La prueba de concordancia de guiones ha sido utilizada en diferentes profesiones de la salud como estrategia para el entrenamiento del RC, sin embargo, no existen estudios que validen su uso en la fonoaudiología, lo que exige el desarrollo de guiones atingentes a la profesión y la necesidad de determinar ciertas propiedades psicométricas de los mismos. El objetivo de esta investigación fue estimar la confiabilidad de un corpus de guiones clínicos diseñado para estudiantes de pregrado de fonoaudiología.

## MÉTODO

Investigación aprobada por el Comité de Ética Científico de la Universidad Santo Tomás, con código 153.20. Cada participante firmó un consentimiento informado para ingresar al proyecto.

Estudio descriptivo de corte transversal. El universo estuvo conformado por profesionales que se desempeñaban en diversos ámbitos clínicos de la fonoaudiología y contaban con un título de fonoaudiólogo, otorgado por una Universidad chilena o extranjera. El muestreo se realizó por conveniencia siguiendo la estrategia de bola de nieve. Los criterios de inclusión fueron: contar con un mínimo de experiencia profesional de 3 años en el área disciplinar, disponer de grado de magíster o doctor y encontrarse habilitado como prestador individual en el Registro Nacional de la Superintendencia de Salud (SIS). Los criterios de exclusión fueron: desempeñarse preferentemente en labores de gestión o docencia (sin actividad clínica) y encontrarse actualmente inactivo en términos laborales debido a jubilación. El número necesario de expertos se seleccionó según la recomendación de la literatura para la validación de guiones clínicos, estimando la incorporación de al menos 10 profesionales por área^(18)^. La carrera intervenida contempla 4 áreas troncales, a saber: Comunicación y Lenguaje Infanto-Juvenil; Voz y Motricidad orofacial; Cognición y Comunicación en Adultos y Personas Mayores; Audiología y Vestibular, por lo que el n mínimo se proyectó en 40 fonoaudiólogos quienes fueron contactados a nivel nacional a través de la técnica de muestreo antes señalada, corroborando una experiencia profesional intermedia y con distribución de género coherente con el perfil profesional de los practicantes en el país.

En términos procedimentales y para la creación del corpus, se convocó a 8 profesionales (2 por área) que redactaron los guiones para el entrenamiento y evaluación de RC en tareas de diagnóstico, estudio e intervención. Dichos expertos recibieron una capacitación sincrónica de 3 horas cronológicas, vía online, a través de la plataforma Microsoft Teams.

La capacitación fue facilitada por un académico con grado de doctor en educación y experiencia aplicada en la metodología, específicamente para la formación médica en pre y posgrado. La instancia fue asesorada por dos fonoaudiólogos con experiencia clínica y académica, pertenecientes al programa de pregrado de origen. Se realizó una exposición teórica, seguida de un taller para la creación de scripts en salas virtuales según área o ámbito formativo. Posteriormente, los productos fueron socializados y retroalimentados por parte del facilitador con objeto de resolver cualquier duda procedimental o teórica, en relación con la confección de guiones. Cada profesional participante de la capacitación tuvo la responsabilidad de crear 10 scripts.

Siguiendo la metodología informada por la literatura, se determinó la población que utilizaría los guiones clínicos^(19)^, en este caso: estudiantes que cursaron los semestres séptimo y octavo de la carrera de fonoaudiología, de la Universidad Santo Tomás, en las sedes Viña del Mar, Santiago y Puerto Montt, durante el año 2021. Para los fines de esta investigación los educandos no utilizaron los guiones, sin embargo, la caracterización de estos es importante dada su relevancia como destinatarios del producto final. La identificación de contenidos clave para el corpus se hizo a través del análisis de resultados de aprendizaje (RA) de programas de asignaturas^(20)^ que tributaban a las cuatro áreas previamente declaradas.

Se creó un corpus de 80 scripts, organizado en las cuatro áreas formativas, es decir, 20 por área. Los guiones de cada área se organizaron como sigue: 8 guiones de diagnóstico, 2 de estudio y 10 de intervención.

Una vez generados los guiones en el formato establecido, se sometieron a revisión por parte del equipo investigador conformado por dos fonoaudiólogos con grado de magíster, experiencia en docencia e innovación educativa, en conjunto al experto con grado de doctor proveniente del área de formación médica, con objeto de homogeneizar los textos y adaptar estos a los fines del estudio.

Para la respuesta y validación de los guiones clínicos (80 en total), cada profesional fonoaudiólogo recibió un cuadernillo con instrucciones y debió responder en un plazo máximo de 2 semanas según su área de expertis.

Las respuestas de los fonoaudiólogos fueron tabuladas en Microsoft Excel y con fines descriptivos se determinó la frecuencia de respuesta por script para luego calcular la puntuación, puntaje transformado y puntaje final según la metodología reportada previamente. Las variables cuantitativas fueron resumidas mediante medidas de tendencia central y dispersión considerando un IC del 95%. De acuerdo con la recomendación de la literatura^(21)^ la consistencia interna se estimó a partir del coeficiente α de Cronbach y la confiabilidad inter observador se calculó con el coeficiente Kappa de Fleiss considerando un nivel de significancia p<0.05^(22)^. Para controlar variables confundentes se estratificaron las respuestas por área, tipo de script y tipo de respuesta. Se eliminó del corpus aquellos guiones incompletos o que presentaran frecuencias de respuesta muy bajas según el método de outliers^(23)^.

El análisis estadístico se realizó en el Stata Statistical Software: Release 15. College Station, TX: StataCorp LLC.software.

## RESULTADOS

Un total de 41 fonoaudiólogos dio respuesta a los cuadernillos entregados por área, con una edad mínima de 28 años y máxima de 61. En cuanto a la distribución según género, se observó un 78% del femenino en el ámbito de comunicación y lenguaje infanto juvenil y un 60% del masculino en voz y motricidad orofacial. El 98% de los informantes contó con grado académico de magíster y el 2% con doctorado. El promedio de experiencia profesional se registró en un rango de 10.6 a 17.2 años (Tabla 1).

**Tabla 1:** Características sociodemográficas del panel de expertos por área

**Área**	**Expertos**	**Edad**			**Experiencia**			**Género**	
	n	Media	Error estándar	Intervalo de confianza 95%	Media	Error estándar	Intervalo de confianza 95%	Masculino	Femenino
**Comunicación y Lenguaje Infanto-Juvenil**	11	40.7	2.56	35 - 46.45	17.18	2.45	11.7 - 22.66	22%	78%
**Voz y Motricidad orofacial**	10	35.4	2.16	30.51- 40.29	11.2	2.22	6.17 - 16.22	60%	40%
**Cognición y Comunicación en Adultos y Personas**	10	34	0.82	32.15 - 35.8	10.6	0.76	8.87 - 12.32	40%	60%
**Audiología y Vestibular**	10	34.8	1.38	31.66 - 37.94	10.6	1.31	7.62 - 13.58	40%	60%

Subtítulo: n = número de participantes

Los puntajes finales agregados y estratificados por área y tipo de script dan cuenta de tendencias de respuesta coherentes entre los profesionales (Figura 1). Se evidencia que la frecuencia de los puntajes agregados suele ajustarse al tipo de respuesta esperada por guión, es decir: positiva, neutra o negativa. En el caso de las áreas de Voz y Motricidad Orofacial, junto a Cognición y Comunicación en Adultos y Personas Mayores se advirtió un desplazamiento hacia valores positivos de las respuestas neutras (Figura 2E, H), para Scripts de tipo estudio, así mismo en el caso de diagnóstico (Figura 2J) e intervención y de los scripts de audiología y vestibular (Figura 1L). En el caso del área Voz y Motricidad Orofacial, de igual modo, hay un desplazamiento de las respuestas negativas hacia la derecha para el caso diagnóstico (Figura 2D) y estudio (Figura 2E) e igual situación se observó en los Scripts de estudio en el área Audiología y Vestibular (Figura 2K).

**Figura 1:** Procedimiento para la validación del corpus de Scripts

Subtítulo: A1 = Comunicación y Lenguaje Infanto-Juvenil; A2 = Voz y Motricidad orofacial; A3 = Cognición y Comunicación en Adultos y Personas Mayores; A4 = Audiología y Vestibular

**Figura 2:** Puntajes finales según área y tipo de Script

Subtítulo: A-C = Comunicación y Lenguaje Infanto-Juvenil; D-F = Área Voz y Motricidad Orofacial; G-I = Cognición y Comunicación en Adultos y Personas; J-L = Área Audiología y Vestibular

La consistencia interna del corpus fue α= 0.67. Al analizar por área y tipo de Script, se observó una confiabilidad baja (α=0.60-0.70) para los ítems diagnósticos en las cuatro áreas, así como para los guiones de intervención en el caso de Cognición y Comunicación en adultos y personas mayores. Cabe destacar que en el caso del área Audiología y Vestibular para los guiones de estudio, se registró una confiabilidad insuficiente (α=0.34). Por otra parte, los Scripts de igual tipo en las áreas restantes, mostraron una confiabilidad aceptable (α=0.80-0.90) (Tabla 2).

**Tabla 2:** Confiabilidad del Corpus según área y tipo de Scripts

**Área**	**Tipo de Script**	**α Cronbach**
**Comunicación y Lenguaje Infanto-Juvenil**	Diagnóstico	0.66
	Estudio	0.84
	Intervención	0.59
**Voz y Motricidad orofacial**	Diagnóstico	0.68
	Estudio	0.82
	Intervención	0.72
**Cognición y Comunicación en Adultos y Personas**	Diagnóstico	0.69
	Estudio	0.82
	Intervención	0.65
**Audiología y Vestibular**	Diagnóstico	0.69
	Estudio	0.34
	Intervención	0.64

Se estimó el acuerdo entre evaluadores en κ= 0.29 (aceptable) para el corpus. Al estratificar según las variables de área y tipo de script, se observó una concordancia leve en las áreas de Voz y Motricidad Orofacial (κ= 0.07), Cognición y Comunicación en Adultos y Personas Mayores (κ= 0.08). El resto de los ítems presentaron un acuerdo aceptable (κ>0.20 y <0.41) y moderado, para los guiones de diagnóstico en Audiología y Vestibular (κ= 0.45) (Tabla 3).

**Tabla 3:** Coeficiente Kappa de Fleiss según área y tipo de Script

**Área**	**Tipo**	**Kappa combinada**	**Z**	**Prob > Z**
**Comunicación y Lenguaje Infanto-Juvenil**	Diagnóstico	0.38	25.43	0.0001
	Estudio	0.25	8.99	0.0001
	Intervención	0.21	13.85	0.0001
**Voz y Motricidad orofacial**	Diagnóstico	0.39	24.42	0.0001
	Estudio	0.07	2.07	0.0193
	Intervención	0.27	19.64	0.0001
**Cognición y Comunicación en Adultos y Personas**	Diagnóstico	0.26	15.51	0.0001
	Estudio	0.08	2.41	0.008
	Intervención	0.17	12.2	0.0001
**Audiología y Vestibular**	Diagnóstico	0.45	22.76	0.0001
	Estudio	0.22	4.27	0.0001
	Intervención	0.22	12.47	0.0001

## DISCUSIÓN

La diversificación de estrategias de enseñanza orientadas al desarrollo de competencias complejas como el RC, resulta clave en la formación de estudiantes de pregrado en salud^(24)^. A la fecha se han reportado experiencias de implementación de la prueba de concordancia de guiones en carreras como medicina y enfermería^(25)^, entre otras. Los resultados de este este estudio dan cuenta de una variabilidad en el juicio de los expertos por área (Figura 1), lo que se alinea con la descripción del proceso de toma de decisiones en escenarios de incertidumbre^(26)^, y resulta coincidente con experiencias previas de investigación donde se han descrito respuestas divergentes como un hallazgo recurrente en la resolución de guiones clínicos por parte de paneles expertos^(23)^.

La evaluación del RC es un proceso complejo en términos pedagógicos^(27)^ y los scripts han demostrado ser una herramienta didáctica confiable y válida cuando se cuenta con un proceso de diseño y construcción riguroso^(28)^. En este sentido es importante destacar que los scripts han permitido discriminar grados de rendimiento diferenciales de acuerdo con el nivel formativo y el tiempo de experiencia profesional en medicina^(28)^, por lo que su aplicación para el posgrado en fonoaudiología representa una proyección interesante que puede explorarse en futuras investigaciones.

El uso de scripts implica ciertos desafíos cuando se piensa en su diseño, construcción e implementación, puntualmente en lo referido a validez de los corpus, al manejo de los bajos puntajes y la asociación de los resultados con otras técnicas de evaluación. En este sentido y para fines prácticos, como se informa en la metodología, se eliminaron del corpus todos los scripts que presentaron respuestas incompletas y a aquellos que no contaron con acuerdos dominantes, es decir una frecuencia considerablemente baja por parte de los expertos^(23)^. Así se obtuvo una confiabilidad global de α= 0.67 que si bien es considerada baja, se asimila a lo reportado en un estudio previo en posgraduados de medicina (α>0.65 en todos los ítems)^(29)^.

La consistencia en las respuestas de los expertos de los guiones en general resulta aceptable más allá del azar (Tabla 3) con excepción de los scripts estudio en las áreas de voz-motricidad orofacial, cognición y Comunicación en adultos y personas mayores. Este hallazgo es coherente con respecto a estudios en otras disciplinas donde se advierte coincidencia en el acuerdo entre los validadores con puntuaciones que suelen ser superiores a las observadas en los novatos sin experiencia y permiten discriminar el nivel formativo, así como la competencia^(30)^. De esta forma se constata que el dossier creado cuenta con propiedades que lo vuelven funcional para evaluar las habilidades de RC en estudiantes de fonoaudiología, tal como la evidencia lo destaca en otras áreas de la salud^(29)^.

Una limitación de este estudio es la variabilidad en el hacer y juicio profesional, lo que se expresa como dispersión en las tendencias de respuesta por parte de los expertos en determinadas áreas (Figura 2D, E, H, J) e impacta en los puntajes de confiabilidad (Tabla 2). Sin embargo, la literatura ofrece algunas explicaciones a este fenómeno como la tendencia de los profesionales validadores a evitar respuestas extremas es decir -2 o +2 en los scripts^(16)^. En esta línea, si bien el puntaje global de confiabilidad resultó bajo, es importante destacar que se mantuvo en el análisis el resultado de confiabilidad pobre (α= 0.34), para el caso de los scripts de estudio en el área audiología y vestibular, lo que afectó el cálculo final sobre el dossier por una parte, pero evitó el sesgo confirmatorio. Con todo, la discrepancia entre los expertos es aceptable, considerando que el instrumento validado no corresponde a un instrumento diagnóstico.

En relación con el tamaño muestral, es importante advertir que se consideró la recomendación de la literatura, por lo que si bien se puede proyectar ampliar el número de expertos por ámbito, esta investigación se apegó a lo sugerido por investigaciones previas^(18)^.

Como proyección resulta interesante incorporar una validación de contenido del dossier, más allá de la adscripción a los resultados de aprendizaje de los programas de asignatura, así mismo sumar una segunda instancia evaluativa para determinar la consistencia de las respuestas intraevaluador, incrementar el número de script por tipo, y aumentar el número de validadores. Así mismo resultará fundamental el determinar el impacto y asociación de la incorporación de esta estrategia con respecto al progreso de los estudiantes más allá de la toma de decisiones clínicas en escenarios con elevada incertidumbre^(31)^.

## CONCLUSIÓN

El corpus creado presentó una confiabilidad global baja, sin embargo, la estratificación por área y tipo de script ofrece un panorama heterogéneo, donde es posible distinguir aquellos ítems donde se obtuvo mayor o menor correlación, según la tarea de razonamiento clínico presentada. Adicionalmente, el acuerdo interevaluador resultó aceptable para todo el dossier. La determinación de propiedades psicométricas de scripts en el contexto de la prueba de concordancia de guiones es una primera aproximación formal a la generación de una herramienta pedagógica innovadora para el entrenamiento y la evaluación auténtica de RC en estudiantes del pregrado de la carrera de Fonoaudiología. Es necesario incrementar la investigación e innovación educativa en este ámbito, con objeto de ofrecer instrumentos formativos pertinentes, que estén basados en la evidencia científica disponible y cuenten con validación para mejorar el desarrollo, seguimiento y evaluación de competencias profesionales en estudiantes de carreras de la salud, ya en instancias preclínicas y/o en etapas formativas tempranas, complementando las estrategias, instrumentos y herramientas ya establecidos en los currículums. La estrategia desarrollada es transferible, pero debe ajustarse a los marcos curriculares de cada casa de estudio en cuanto a contenidos y resultados de aprendizaje.
